# Pressure-flow discordance in perioperative pulmonary hypertension: a mechanism-based review and provisional five-domain right-heart model for noncardiac surgery

**DOI:** 10.3389/fmed.2026.1972194

**Published:** 2026-09-28

**Authors:** Budanbaila La, Yanyan Bai, Yiri Du, Yi Qiu, Ling Wang

**Affiliations:** 1 Department of Anesthesiology, The Affiliated Hospital of Inner Mongolia Medical University, Hohhot, China; 2 Department of Anesthesiology, Hohhot Maternal and Child Health Hospital, Hohhot, China; 3 Department of Anesthesiology, The Second Affiliated Hospital of Inner Mongolia Medical University, Hohhot, China

**Keywords:** hemodynamics, noncardiac surgery, perioperative medicine, pressure-flow discordance, pulmonary hypertension, right ventricle, right ventricular failure, ventricular-arterial coupling

## Abstract

Pulmonary hypertension increases perioperative risk because anesthesia, positive-pressure ventilation, bleeding, and surgical stress can rapidly disturb coupling between the right ventricle and the pulmonary circulation. Pulmonary artery pressure alone cannot distinguish successful unloading from loss of right-ventricular output. This narrative review integrates contemporary guidance, noncardiac surgical cohorts, right-heart physiology, echocardiography, and critical-care evidence and then proposes a provisional five-domain model. The domains are pulmonary vascular load; right-ventricular reserve and ventricular-arterial coupling; systemic perfusion; effective preload and venous congestion; and forward flow and oxygen delivery. The underlying physiology and most component management principles are established; the novel contribution is their longitudinal organization into pressure-flow trajectories, provisional dominant phenotypes, and timestamped intervention-response pairs. Evidence provenance is labeled as guideline or consensus, direct perioperative clinical data, extrapolated physiologic or critical-care evidence, or an unvalidated framework hypothesis. A measurement hierarchy separates quantitative cardiac-output estimates from stroke-volume trends and from downstream perfusion surrogates. Provisional classification requires a core directional pattern plus corroboration from independent domains and permits mixed or indeterminate states. Disease group remains decisive because the same trajectory may imply different hazards in precapillary, postcapillary, lung-disease, or thromboembolic pulmonary hypertension. During controlled instability, one small matched intervention can test the working mechanism; during immediately life-threatening deterioration, parallel resuscitative actions take precedence. The proposed model must demonstrate reproducible classification and incremental value beyond contemporary clinical risk assessment, right-ventricular function, biomarkers, procedure characteristics, and conventional monitoring before it is used as a treatment algorithm.

## 1 Introduction

Pulmonary hypertension (PH) is a hemodynamic condition rather than a single disease. The contemporary definition is a resting mean pulmonary artery pressure (mPAP) greater than 20 mmHg. Precapillary PH additionally requires a pulmonary artery wedge pressure (PAWP) of 15 mmHg or less and pulmonary vascular resistance (PVR) greater than 2 Wood units (1, 2). Pulmonary arterial hypertension (PAH) is World Health Organization (WHO) group 1 PH; PH associated with left-heart disease, lung disease, chronic thromboembolic disease, and mixed or unclear mechanisms follows different biology and treatment pathways (3–6). PAH should not be used as a synonym for all PH. An intervention that increases pulmonary blood flow may unload the RV in precapillary disease but precipitate pulmonary edema when left-sided filling pressure is elevated.

Perioperative danger does not arise from high pulmonary pressure alone. Anesthetic vasodilation lowers aortic pressure. Positive-pressure ventilation alters venous return, transmural pressure, and pulmonary vascular load. Hypoxemia, hypercapnia, and acidemia intensify pulmonary vasoconstriction, whereas hemorrhage reduces effective preload and oxygen-carrying capacity. Excess fluid increases RV wall stress, tricuspid regurgitation, and venous congestion. These disturbances can amplify one another within minutes. International statements therefore emphasize disease phenotyping, RV assessment, continuity of PH-targeted therapy, avoidance of reversible PVR triggers, maintenance of systemic perfusion, and access to advanced rescue (7–9). The unresolved bedside problem is not whether these principles matter, but how to determine which mechanism dominates when several variables move in opposite directions.

Outcome studies confirm substantial but heterogeneous risk. In a prospective international PAH survey, major complications occurred in 6.1% of procedures and 30-day mortality was 3.5%, with emergency surgery carrying the greatest hazard (10). Single-center and administrative cohorts have reported higher rates of heart failure, respiratory failure, hemodynamic instability, prolonged ventilation, readmission, and death among patients with PH undergoing noncardiac surgery (11–14). A multidomain perioperative score showed that patient vulnerability and procedural stress provide complementary information (15). These studies support careful risk stratification, but they do not establish a single pressure threshold that separates compensation from failure.

The observation that PAP may decrease when an exhausted RV can no longer generate pressure is established physiology, not a newly discovered phenomenon. Likewise, RV reserve, preload responsiveness, venous congestion, systemic pressure, cardiac output, and treatment response already appear in AHA, ISHLT, BJA, ESC/ERS, and POQI guidance (2, 7–9, 16, 17). The proposed novelty is narrower: to formalize those familiar elements as a time-updated pressure-flow representation, to separate continuous measurements from provisional bedside labels, and to treat a timestamped intervention-response pair as an auditable unit. Whether that organization improves reliability, prediction, or care is unknown and must be tested.

This review therefore has two linked aims. It first summarizes established perioperative PH and RV physiology with explicit evidence boundaries. It then presents an authors’ framework consisting of five coupled domains, five provisional dominant phenotypes, a two-track decision process, and falsifiable predictions. The framework supplements disease classification, contemporary risk assessment, specialist PH care, and guideline-based management. It is not a guideline, a validated score, or a mandate to treat a phenotype label.

## 2 Review scope, evidence boundaries, and model development

This narrative review considered literature available through 31 July 2026. PubMed/MEDLINE was searched from inception with five concept blocks covering: PH and noncardiac surgery; perioperative or critical-care RV failure and RV-pulmonary arterial coupling; pulmonary pressure paired with cardiac output or stroke-volume signals; vasoactive, pulmonary vasodilator, fluid, decongestion, and extracorporeal interventions; and group-specific PH modifiers. ESC/ERS, AHA/ACC, ISHLT, American Society of Echocardiography, POQI, and relevant journal or society repositories were also searched. Reference lists of pivotal statements and reviews were backward-screened. Complete reproducible search strings and source dates are provided in Supplementary Table S1.

Eligible material included adult PH definitions and classifications; perioperative statements or cohorts; RV-pulmonary vascular physiology; validated or widely used hemodynamic and echocardiographic measurements; acute RV failure, cardiac-surgery, and critical-care evidence when direct noncardiac PH evidence was unavailable; and methodological literature relevant to model validation. Pediatric, obstetric, and isolated congenital-heart studies were excluded unless they supplied indispensable general physiology. Case reports, unreferenced opinion, and treatment claims without a traceable evidence basis were not used to support management statements. Selection was iterative and consensus-based rather than formal duplicate systematic-review screening. A contemporaneous de-duplication and screening log was not retained; retrospective screened-record counts were therefore not reconstructed. The final synthesis contains 51 sources: 20 guidance, consensus, or scientific-statement sources; 6 direct noncardiac perioperative PH outcome or risk studies; 12 physiology, imaging, or monitoring sources; 12 treatment or rescue sources largely extrapolated from cardiac surgery or critical care; and 1 narrative-review methods source. This limitation is stated to avoid a false PRISMA-like precision.

Guidance and scientific statements received the greatest interpretive weight, followed by direct perioperative PH studies, then mechanistic or measurement evidence, and finally indirect treatment evidence from acute RV failure, cardiac surgery, or critical care. SANRA guided reporting (18). Evidence was not pooled because populations, PH groups, procedures, monitoring, interventions, and outcome definitions were too heterogeneous to support a single treatment effect for this framework.

To make provenance visible, four source labels are used. G denotes guideline, consensus, or scientific-statement support. D denotes direct perioperative clinical data in patients with PH undergoing noncardiac surgery; this evidence is predominantly observational. E denotes extrapolated physiologic, measurement, acute-RV-failure, cardiac-surgery, or critical-care evidence. H denotes an authors’ framework hypothesis that requires prospective validation. These labels identify provenance, not certainty: a G statement may still rest on expert consensus, and a D study may be confounded. No H-labeled phenotype, threshold, or phenotype-treatment pairing should be treated as validated practice. Supplementary Table S2 maps the major claims to these labels and their limits.

Model construction was constrained to signals observable with routine or selectively advanced perioperative tools. A proposed phenotype had to explain a potentially harmful reflex treatment or alter the initial direction of assessment. Benefit was defined as a coherent trajectory across pressure, quantitative or directional flow, RV geometry, gas exchange, and congestion rather than correction of one number. The five domains are a data structure; the provisional phenotype is only a concise working summary. Table 1 separates established content, reorganized content, and genuinely new testable propositions.

**Table 1:** Established evidence, reorganized content, and new testable propositions.

Layer	Content	Evidence source	Boundary
Established physiology	PAP reflects load, downstream pressure, and RV-generated flow; PAP may fall when RV output collapses.	E	Not claimed as a new discovery.
Established guidance	Define WHO group, assess RV function, continue PH therapy, avoid reversible PVR triggers, preserve systemic perfusion, use judicious fluid, and plan rescue.	G; D for risk estimates	Recommendations retain disease- and context-specific caveats.
Reorganized representation	Pair pressure with quantitative or directional flow and read the pair with RV geometry, congestion, gas exchange, and systemic perfusion over time.	H, informed by G/D/E	Added clinical value has not been established.
Provisional classification	Five dominant phenotypes with explicit mixed and indeterminate states and a core-plus-two-corroborating-features rule.	H	Requires Delphi refinement and interrater testing.
Auditable decision unit	Timestamped trigger-intervention-response pair; parallel bundle-response record during immediate crisis.	H	Must not delay standard resuscitation.
Testable claim	Incremental prediction and decision value beyond contemporary risk assessment and conventional monitoring.	H	Framework should be narrowed or rejected if value, reliability, or feasibility is absent.

D, direct noncardiac perioperative PH clinical data; E, extrapolated physiologic/measurement/critical-care/cardiac-surgery evidence; G, guideline/consensus/scientific statement; H, authors’ hypothesis requiring validation. Labels indicate provenance, not certainty.

## 3 Why pulmonary artery pressure can mislead

### 3.1 Pressure is generated by both load and flow

The resistive component of RV afterload is expressed as PVR = (mPAP − PAWP)/cardiac output. mPAP therefore depends on pulmonary vascular properties, downstream pressure, and the amount of blood the RV ejects. PVR is incomplete because pulsatile load is influenced by pulmonary arterial compliance, characteristic impedance, wave reflection, heart rate, and ejection timing (19, 20). Two patients with similar PVR can impose different energetic demands on the RV, and one patient can move between states without a proportional change in mPAP.

Acute pulmonary vascular load rises with hypoxemia, hypercapnia, acidemia, sympathetic activation, pulmonary embolic obstruction, high airway pressure, dynamic hyperinflation, and lung volumes at either extreme. Atelectasis promotes hypoxic vasoconstriction and can increase extra-alveolar vessel resistance; overdistension compresses alveolar vessels and raises intrathoracic pressure. A change in PAP has clinical meaning only when interpreted with flow, PAWP or an estimate of left-sided filling, lung mechanics, oxygenation, and RV response.

### 3.2 The pressure-flow quadrant

Figure 1 arranges four directional patterns. High or rising PAP with preserved flow may reflect a coupled RV that is still tolerating the added load. When pressure is high and flow falls, active strain or emerging uncoupling becomes more likely. Lower pressure with rising flow is consistent with true unloading or recovery. The easily missed danger is lower pressure with falling flow, when the RV may no longer generate either adequate pressure or output. These patterns describe change from an individual baseline; they are not universal cutoffs and do not, by themselves, establish a diagnosis.

**Figure 1:**
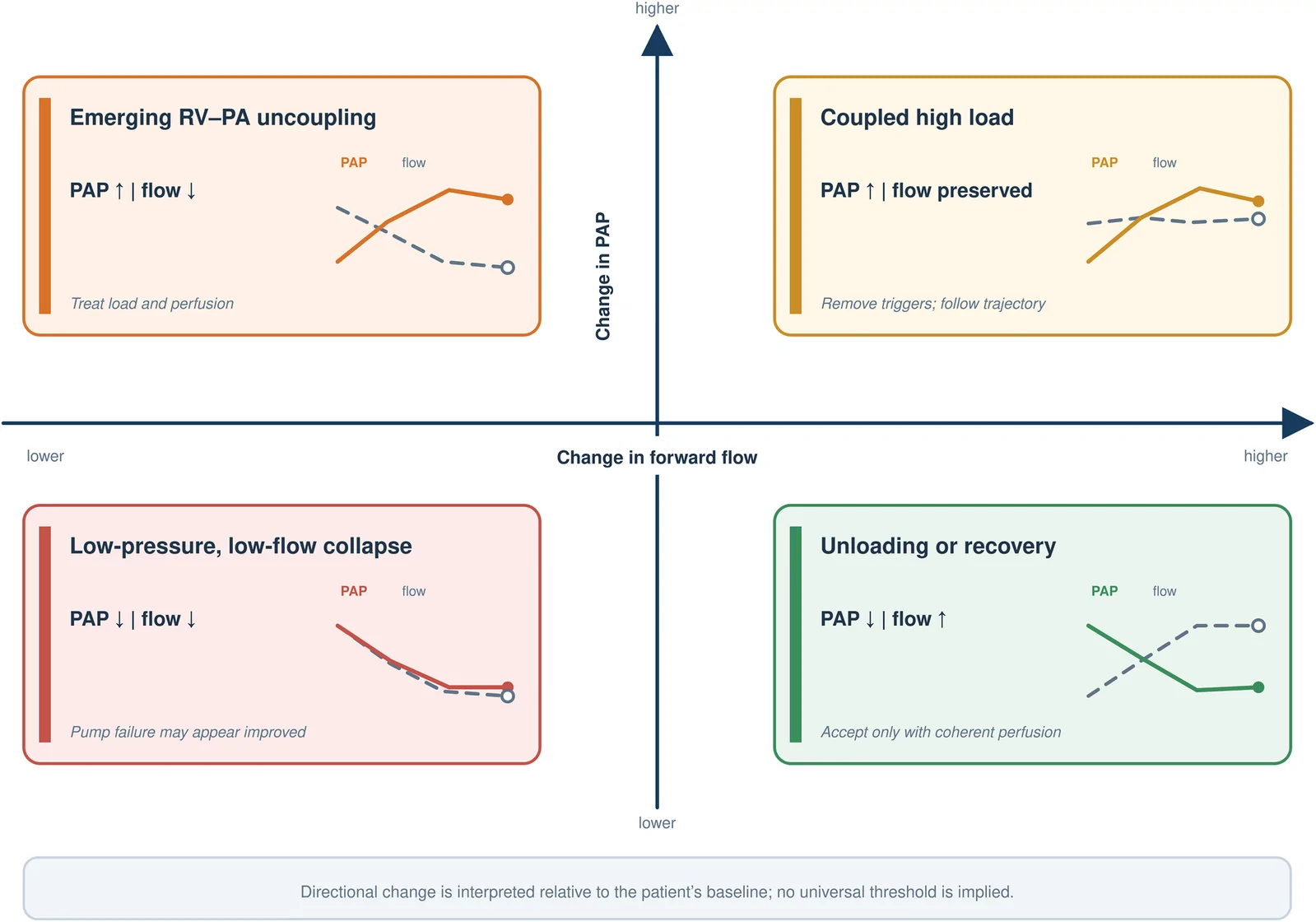
Pressure-flow discordance matrix. Directional changes are interpreted relative to a patient-specific baseline. A fall in pulmonary artery pressure can represent recovery when quantitative or directional flow rises or circulatory collapse when flow falls. PAP, pulmonary artery pressure; RV, right ventricle.

### 3.3 A hierarchy for forward-flow assessment

Forward flow should not be inferred from a collection of interchangeable surrogates. The preferred tier is a serial quantitative cardiac-output estimate obtained with a method suitable for the patient and interpreted within its known limitations. Pulmonary-artery-catheter thermodilution and direct Fick are reference methods but remain estimates and can be distorted by severe tricuspid regurgitation, shunt, injectate technique, unstable oxygen consumption, or rapid hemodynamic change. Calibrated pulse-contour and echocardiographic cardiac output are quantitative estimates whose accuracy depends on calibration, vascular tone, LVOT measurement, Doppler alignment, rhythm, and loading conditions.

The second tier is a same-modality stroke-volume trend, most commonly beat-averaged LVOT VTI or a validated pulse-contour stroke-volume signal. VTI is not cardiac output unless LVOT area and heart rate are incorporated. It can be useful directionally when the same window, beat selection, rhythm, ventilation, and operator technique are maintained. For this proposed framework, a change of at least 15% in quantitative cardiac output or beat-averaged VTI is a provisional measurement rule intended to exceed ordinary repeatability error; it is not a validated biological threshold. Sites should substitute a larger threshold when local repeatability is poorer.

The third tier comprises supportive directional signals. End-tidal carbon dioxide may track pulmonary blood flow only when minute ventilation, metabolic production, dead space, airway mechanics, and ventilation-perfusion matching are sufficiently stable. SvO2 and ScvO2 reflect the balance among flow, oxygen consumption, hemoglobin, and arterial saturation. Lactate, urine output, capillary refill, skin temperature, and mental status are downstream perfusion or oxygen-delivery signals, not measurements of flow, and their time constants differ. A pressure-flow classification should be called quantitative only when a tier-1 estimate is available, directional when tier-2 evidence is available, and supportive or indeterminate when it rests on tier-3 findings alone. Table 2 summarizes this hierarchy.

**Table 2:** Hierarchy for forward-flow and downstream assessment.

Tier	Examples	Principal confounders	Permitted interpretation
1. Serial quantitative cardiac-output estimate	PAC thermodilution or direct Fick; calibrated pulse contour; echocardiographic CO when LVOT area and heart rate are measured	Method-specific error; severe TR or shunt; unstable VO2; calibration and vascular tone; LVOT diameter and Doppler alignment; rhythm	Use as primary flow evidence when technically valid; no routine method is literally direct flow.
2. Same-modality stroke-volume trend	Beat-averaged LVOT VTI; validated stroke-volume trend	Same window and operator; beat selection; rhythm; ventilation; loading conditions	Directional flow evidence. A ≥15% change is an H-labeled feasibility rule, not a biological threshold.
3. Supportive cardiopulmonary signal	ETCO2 under stable ventilation and metabolism	Minute ventilation, dead space, V/Q mismatch, embolism, temperature, metabolic production	Supportive only; downgrade to indeterminate when confounders change.
4. Oxygen-balance signal	SvO2 or ScvO2	VO2, hemoglobin, arterial saturation, extraction, sampling site	Not a flow measurement; use with independent pressure and flow evidence.
5. Downstream perfusion/organ signal	Lactate trend, urine output, capillary refill, skin temperature, mental status	Delayed kinetics, drugs, sepsis, liver or renal function, baseline organ reserve	Confirms consequence or recovery; cannot determine beat-to-beat flow direction.

CO, cardiac output; ETCO2, end-tidal carbon dioxide; LVOT, left-ventricular outflow tract; PAC, pulmonary artery catheter; ScvO2, central venous oxygen saturation; SvO2, mixed venous oxygen saturation; TR, tricuspid regurgitation; V/Q, ventilation-perfusion; VTI, velocity-time integral; VO2, oxygen consumption.

### 3.4 RV reserve and ventricular-arterial coupling

The normal RV is a thin-walled pump adapted to a low-impedance circulation. Chronic pressure load may initially produce hypertrophy and augmented contractility, but sustained stress can progress to dilation, fibrosis, ischemia, tricuspid regurgitation, and adverse ventricular interaction (21–25). Ventricular-arterial coupling expresses whether RV contractility matches arterial load. The invasive reference is the ratio of end-systolic elastance to effective arterial elastance (Ees/Ea); a falling ratio indicates that the ventricle is spending more energy for less effective stroke work (26).

Pressure-volume loops are rarely available perioperatively. Echocardiographic surrogates such as TAPSE/PASP, fractional area change, tissue Doppler S’, RV free-wall strain, three-dimensional ejection fraction, and cardiac magnetic resonance measures provide complementary information (27–29). Each is load-, rhythm-, geometry-, and image-dependent. The model treats reserve as a latent property inferred from concordant findings rather than as one threshold.

### 3.5 The diastolic pressure-right atrial pressure difference is contextual, not coronary perfusion pressure

A pressure-loaded RV becomes vulnerable when systemic pressure falls while right-atrial pressure (RAP) and wall stress rise. The difference between systemic diastolic pressure and RAP can flag this adverse context, but it is not a direct or complete measure of RV coronary perfusion and is not assigned a treatment threshold in this review. Coronary flow is phasic. RV perfusion can occur during systole and diastole, and its pattern changes as RV systolic and diastolic pressures rise. Local intramyocardial pressure, aortic pressure throughout the cardiac cycle, coronary anatomy, ventricular interaction, heart rate, oxygen content, and metabolic demand all matter. RAP is only an imperfect surrogate for downstream and intramyocardial pressure; positive intrathoracic pressure and waveform timing further complicate interpretation.

The proposed signal is therefore H-labeled and hypothesis-generating. It may support a provisional perfusion-pressure/RV ischemic phenotype only when low or falling systemic pressure, impaired flow, and independent evidence of RV strain or ischemia coexist. A pressure response to a vasopressor can support the working mechanism but cannot validate the pressure-RAP difference as a coronary perfusion measure.

## 4 The five coupled physiologic domains and disease-group modifiers

The five domains are continuous measurements and contextual observations, not mutually exclusive categories (Figure 2). Treatment may improve one domain while impairing another, so any meaningful change requires system-wide reassessment. To reduce cognitive load, the bedside minimum is a pressure-flow pair plus three modifiers: RV geometry or function, congestion or preload response, and gas exchange. Additional variables refine rather than redefine that core.

**Figure 2:**
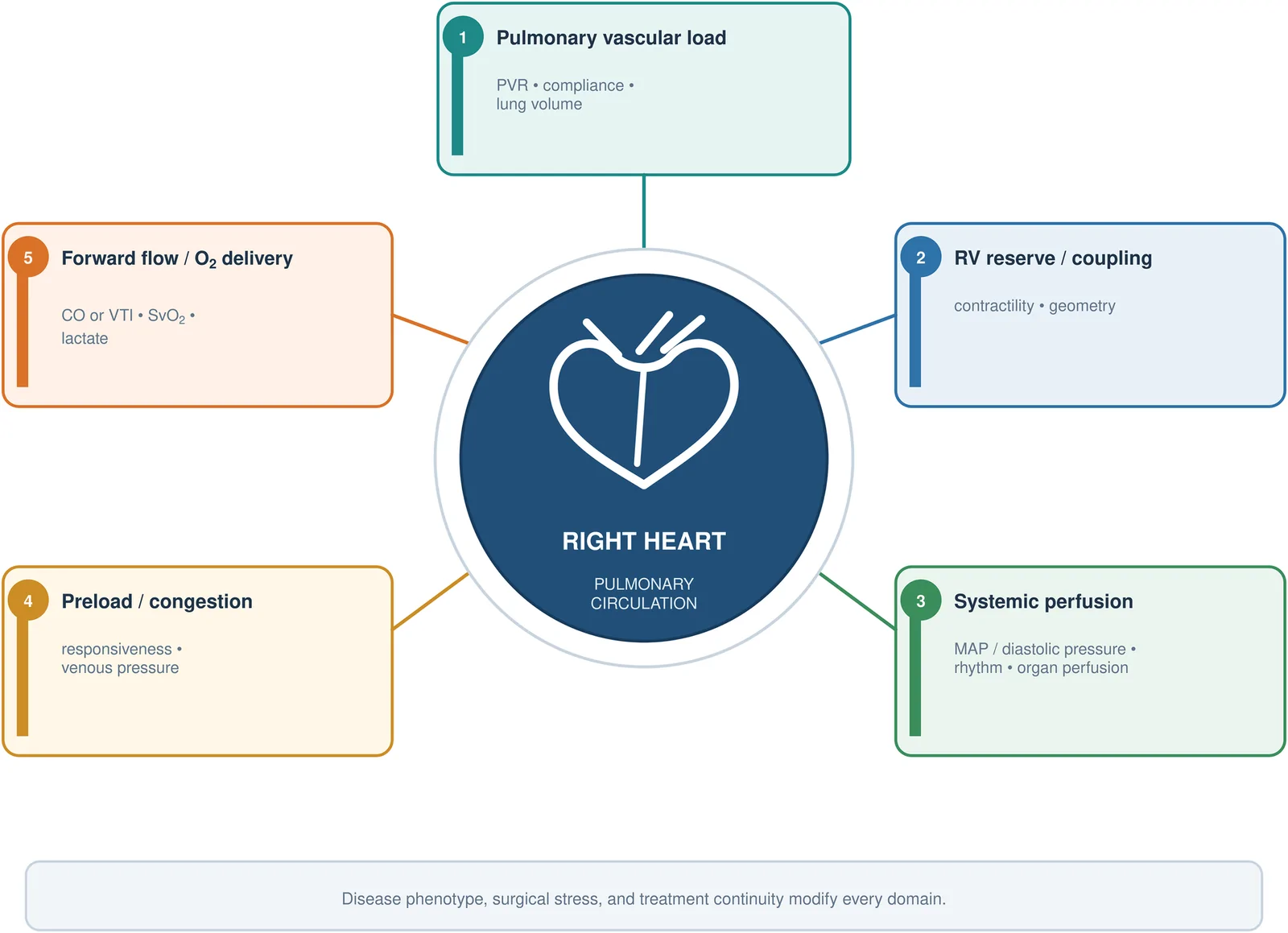
Five-domain right-heart model. Pulmonary vascular load, RV reserve and coupling, systemic perfusion, effective preload and venous congestion, and forward flow and oxygen delivery interact continuously. Disease group, procedural stress, and treatment continuity modify every domain.

### 4.1 Domain 1: pulmonary vascular load

Pulmonary vascular load is assessed by asking whether resistance to RV ejection has risen and why. Relevant signals include oxygenation, PaCO2, pH, airway, plateau, and driving pressures, auto-PEEP, lung volume and compliance, PAP and PVR when available, and septal geometry. Hypoxic vasoconstriction, hypercapnia, embolic obstruction, high intrathoracic pressure, and left-sided congestion call for different responses. Equating mPAP with total afterload, or assuming that every PEEP change has the same effect, obscures that distinction.

### 4.2 Domain 2: RV reserve and coupling

RV reserve describes whether the ventricle can match the current load. Assessment can integrate RV/LV size, fractional area change, TAPSE, tissue Doppler S’, RV strain, tricuspid regurgitation, septal motion, and the flow trajectory. Absolute thresholds characterize risk but should not become universal clearance rules. A chronically adapted RV may tolerate a modest pressure increase, whereas a dilated, ischemic, or rhythm-dependent RV can fail after a smaller new load.

### 4.3 Domain 3: systemic perfusion

Systemic mean and diastolic pressure, rhythm, ECG changes, capillary refill, mentation, urine output, lactate, and RV geometry help show whether perfusion is failing. The domain is deliberately named systemic perfusion rather than right-coronary perfusion because the latter is not measured directly. Reducing PVR while systemic hypotension persists may worsen RV ischemia and contractility.

### 4.4 Domain 4: effective preload and venous congestion

Venous return is essential, yet preload dependence does not establish fluid responsiveness. CVP is shaped by intrathoracic pressure, RV compliance, tricuspid regurgitation, waveform timing, and volume; no fixed CVP defines adequate filling. Passive leg raising, a mini-fluid challenge, same-modality stroke-volume or VTI response, RV geometry, hepatic or portal venous Doppler, edema, and organ function help distinguish underfilling from congestion. The practical question is whether a small preload change increases useful flow without enlarging the RV or raising venous pressure further.

### 4.5 Domain 5: forward flow and oxygen delivery

Forward flow and oxygen delivery show whether treatment restores useful systemic perfusion. Quantitative cardiac-output estimates and same-modality stroke-volume trends carry more weight than ETCO2, venous oxygen saturation, lactate, urine output, or clinical perfusion. Arterial pressure remains necessary but cannot stand in for flow. Nor is a higher measured output an unqualified success if achieved at the cost of severe tachycardia, systemic hypotension, or worsening oxygenation.

### 4.6 Applicability across WHO groups and perioperative settings

The five-domain data structure can be recorded across PH groups, but interpretation and therapy remain disease-specific. In group 1 PAH and other precapillary states, selective reduction of pulmonary vascular tone may improve RV ejection when systemic pressure can support it. In group 2 PH, increased pulmonary blood flow can raise left-atrial pressure and precipitate pulmonary edema; left-sided filling, mitral disease, and LV compliance must therefore modify interpretation. In group 3 PH, pulmonary vasodilation may worsen ventilation-perfusion matching and hypoxemia even when RV load falls. In group 4 chronic thromboembolic disease, fixed obstruction, residual or recurrent thrombus, anticoagulation, and access to pulmonary endarterectomy or balloon pulmonary angioplasty expertise shape the response. Group 5 requires cause-specific judgment because mechanisms are heterogeneous (2–6).

Accordingly, a phenotype label never authorizes a drug independently of WHO group, pre- versus postcapillary hemodynamics, gas exchange, and procedural context. Table 3 shows the principal modifiers. The model is best described as an RV-focused observation framework with disease-specific interpretation, not a universal PH treatment pathway.

**Table 3:** WHO group and hemodynamic modifiers of five-domain interpretation.

PH group/type	Dominant biological context	Interpretation modifier	Safety risk
Group 1 PAH/precapillary	High fixed or reactive PVR; RV-PA uncoupling and treatment interruption	Selective pulmonary vasodilation may improve ejection if systemic pressure and LV filling are adequate	Systemic hypotension, rebound after interruption, delayed rescue
Group 2 PH/postcapillary	Elevated left-sided filling pressure; RV and LV interaction; congestion	A flow rise may increase LA pressure; assess LV/valves/PAWP and pulmonary edema before vasodilation or fluid	Pulmonary edema from increased flow; indiscriminate PAH therapy
Group 3 PH/lung disease	Hypoxic vasoconstriction, parenchymal disease, limited ventilatory reserve	Interpret PAP and ETCO2 with V/Q matching, dead space, oxygenation, and airway pressure	Worsened V/Q mismatch or hypoxemia despite reduced PAP
Group 4 CTEPH/CTEPD	Fixed or residual obstruction, acute-on-chronic embolic risk, anticoagulation	Consider obstruction and anticoagulation; coordinate with CTEPH intervention expertise	Missing acute embolism, interruption/bleeding trade-off, delayed definitive referral
Group 5/mixed	Multifactorial or unclear mechanisms	Use five-domain observations only after cause-specific assessment; avoid assuming a PAH-like response	False unification of heterogeneous disease

CTEPD, chronic thromboembolic pulmonary disease; CTEPH, chronic thromboembolic pulmonary hypertension; LA, left atrial; LV, left ventricle; PAH, pulmonary arterial hypertension; PAWP, pulmonary artery wedge pressure; PVR, pulmonary vascular resistance.

## 5 Provisional dominant perioperative phenotypes

The domains describe physiology; a phenotype names the mechanism most likely to guide the next assessment or initial direction at a particular time. The phenotypes are neither discrete diseases nor obligatory stages (Figure 3). They can overlap, reverse, or remain indeterminate after induction, ventilation, bleeding, positioning, pneumoperitoneum, analgesia, or treatment. Clinicians should record one dominant phenotype only when the provisional core criterion and at least two corroborating features from independent domains are present. Signals derived from the same measurement should not be counted twice.

**Figure 3:**
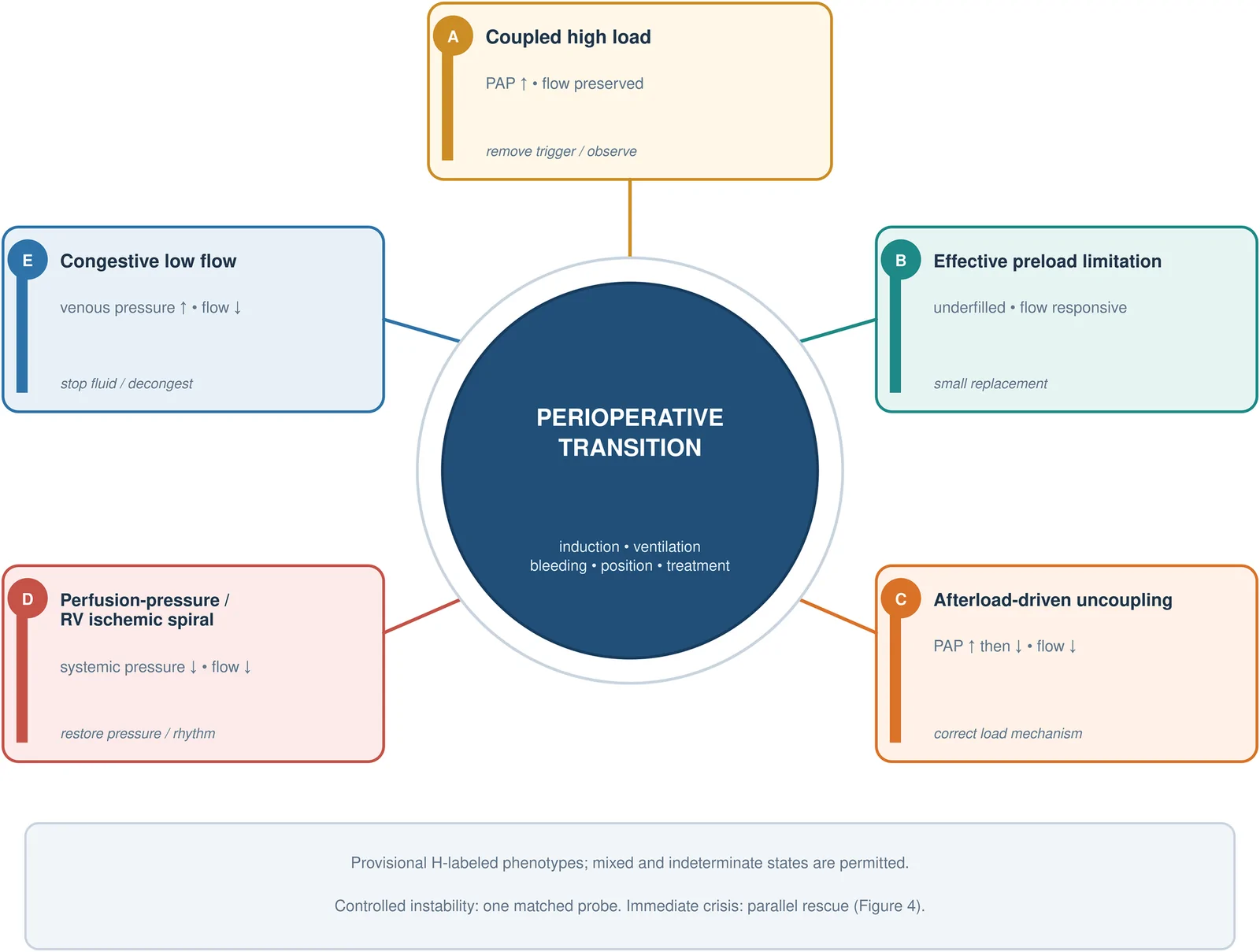
Five provisional dominant perioperative phenotypes and potential transitions. Phenotypes can overlap and may change after induction, positive-pressure ventilation, bleeding, positioning, vasoactive therapy, or fluid. Mixed and indeterminate states should be recorded rather than forced into one category.

A change in quantitative cardiac output or beat-averaged VTI of at least 15% is used as the provisional definition of rising or falling flow, provided the same method is used under sufficiently stable rhythm and ventilatory conditions. New or worsening RV dilation may be recorded when the RV/LV basal-diameter or end-diastolic area ratio increases by at least 0.2 or crosses 1.0, or when a clearly new septal-flattening pattern appears. These values are H-labeled feasibility rules chosen for reproducible testing, not validated treatment thresholds. If measurement variability exceeds the proposed change, the state is indeterminate.

When competing phenotypes meet criteria, both should be recorded and the time-critical reversible threat should determine the immediate action. A patient with high RAP, RV dilation, hypotension, and a positive preload response is not forced into one box: the state is recorded as mixed congestive/preload-recruitable, only a small response-guided volume is considered if there is no life-threatening crisis, and congestion is reassessed after each increment. If evidence is insufficient or contradictory, the correct label is indeterminate and management should address observable physiology rather than the label. Table 4 provides the operational rules; Supplementary Table S4 provides the coding manual.

**Table 4:** Provisional operational criteria for dominant perioperative phenotypes.

Phenotype	Provisional core and corroborating criteria	Exclusions or mixed-state rule	Initial direction and evidence boundary
Coupled high load	Load/PAP high or rising; quantitative/directional flow stable or rising; plus ≥2: stable systemic perfusion, stable RV geometry, preserved oxygen delivery	New RV dilation/septal flattening or flow fall shifts toward uncoupling; insufficient flow data = supportive/indeterminate	Reverse triggers; do not treat PAP alone. Components G/E; classification H.
Effective preload limitation	≥15% flow rise with PLR or mini-fluid under stable conditions; plus ≥2: plausible volume/venous-return loss, small/normal RV or no geometric worsening, no progressive venous congestion	High RAP/dilated RV with positive response = mixed congestion/preload-recruitable; reassess after every increment	Control bleeding/impediment; small responsive replacement. Components G/E; 15% rule and phenotype H.
Afterload-driven RV-PA uncoupling	≥15% flow fall; new/worsening RV dilation or septal flattening; plus an acute load trigger and ≥1 independent perfusion/congestion change	PAP may rise early or fall late; predominant systemic hypotension with pressor response may favor ischemic spiral	Correct trigger; consider selective inhaled therapy only with group-specific safeguards. Components G/E; phenotype H.
Perfusion-pressure/RV ischemic spiral	Systemic/diastolic pressure falls with ≥15% flow fall; plus ≥2: high/rising RAP, RV dilation, ischemic ECG/echo clue, relevant dysrhythmia	Diastolic pressure-RAP difference is not coronary perfusion pressure; no validated threshold	Restore systemic pressure/rhythm and reassess flow. Components G/E; composite phenotype H.
Congestive low flow	≥15% flow fall; high/rising venous pressure or congestion; no ≥15% flow response to preload; plus ≥1: RV dilation/TR, abnormal venous Doppler, organ dysfunction	Positive preload response does not exclude congestion; record mixed state when both are present	Stop nonresponsive fluid; address load/contractility; controlled decongestion. Components G/E; phenotype H.

All classification rules and thresholds are H-labeled and unvalidated. A dominant label requires the core pattern plus at least two independent corroborating features unless the row specifies otherwise. Use the same flow modality under stable conditions. Mixed and indeterminate states are valid classifications. PA, pulmonary arterial; PAP, pulmonary artery pressure; PLR, passive leg raise; RAP, right-atrial pressure; RV, right ventricle; TR, tricuspid regurgitation.

### 5.1 Coupled high-load phenotype

PAP or another load signal is elevated while quantitative or directional flow, systemic perfusion, RV geometry, and oxygen delivery remain preserved. The RV is working harder, but new failure is not demonstrated. Management focuses on reversible load triggers and avoids escalation directed only at the pressure number. A pressure decrease counts as benefit only when flow and perfusion remain stable or improve. Evidence boundary: trigger avoidance and preservation of RV/systemic physiology are G/E; the named phenotype and its classification rule are H.

### 5.2 Effective preload-limited phenotype

Hemorrhage, venodilation, excessive mean intrathoracic pressure, caval compression, or another mechanical limit to venous return can produce this state. The core criterion is a reproducible flow rise of at least 15% during passive leg raising or a small, rapidly reassessed preload challenge under stable measurement conditions. Treatment addresses bleeding or the mechanical impediment, then replaces volume or blood products in response-guided increments. Fluid responsiveness does not exclude simultaneous congestion. Evidence boundary: assessment of fluid responsiveness and avoidance of indiscriminate boluses are G/E; the phenotype rule and its pairing with the full five-domain vector are H.

### 5.3 Afterload-driven RV-pulmonary arterial uncoupling

This phenotype requires falling flow plus new or worsening RV strain or geometry and an acute load trigger such as hypoxemia, hypercapnia, acidemia, embolic obstruction, overdistension, dynamic hyperinflation, or increased pulmonary vascular tone. PAP may rise early and fall later as contractile reserve is exhausted. Treatment begins with the trigger. A selective inhaled pulmonary vasodilator may be considered when systemic pressure is supportable and left-sided filling and gas-exchange risks have been assessed. Evidence boundary: correction of reversible PVR triggers is G/E; inhaled therapy evidence in noncardiac PH surgery is limited and is largely E; the operational phenotype is H.

### 5.4 Perfusion-pressure/RV ischemic spiral

The core pattern is low or falling systemic and diastolic pressure with falling flow, accompanied by high or rising RAP, RV dilation, ischemic evidence, or a relevant dysrhythmia. PAP may remain unchanged or fall. The systemic diastolic pressure-RAP difference is contextual only and is not treated as RV coronary perfusion pressure. Restoration of systemic pressure and rhythm may support the working mechanism when followed by coherent flow and geometric improvement. Evidence boundary: preservation of systemic pressure and rhythm is G/E; the named phenotype, composite signal, and any future threshold are H.

### 5.5 Congestive low-flow phenotype

This state combines falling flow with high or rising venous pressure or other congestion evidence and no meaningful flow response to preload. RV dilation, significant tricuspid regurgitation, septal shift, abnormal systemic venous Doppler, edema, and worsening renal or hepatic function provide support. Treatment stops nonresponsive fluid, preserves systemic perfusion, addresses afterload and contractility, and may include carefully titrated decongestion. Evidence boundary: avoidance of nonresponsive fluid and management of RV failure are G/E; the phenotype and its operational criteria are H.

## 6 Applying the model across the perioperative journey

### 6.1 Preoperative evaluation: reserve relative to the proposed insult

Perioperative risk reflects patient reserve, procedural stress, and the ability to rescue deterioration. The decision begins with whether surgery is needed now, whether a less invasive option can achieve the same goal, and whether the procedure belongs in a PH-capable center. Elective delay is useful only when it permits a defined intervention, such as treatment of infection or anemia, correction of hypoxemia or an acid-base disorder, relief of congestion, rhythm control, or optimization of disease-targeted therapy. An open-ended search for a lower PAP offers little value.

The minimum preoperative assessment includes WHO group and hemodynamic type; cause and trajectory of PH; targeted therapy and interruption risk; WHO functional class; symptoms of RV failure; recent echocardiography; natriuretic peptide when clinically relevant; oxygen requirement; rhythm; anticoagulation; prior decompensation; and planned procedural stress. Current statements support a multidisciplinary plan for high-risk patients (7–9, 16, 17, 30, 31). Right-heart catheterization is not routine but may be reasonable when phenotype remains uncertain, noninvasive and clinical findings conflict, or invasive information would change surgery, targeted therapy, or rescue planning.

The authors’ framework adds an H-labeled baseline snapshot across the five domains and anticipates the most plausible transition. A patient with severe PAH, preserved flow, and stable RV geometry may begin with coupled high load yet remain vulnerable to systemic hypotension during induction. A patient with group 2 PH and congestion may be threatened more by fluid and pulmonary edema than by a modest pressure increase. This anticipated transition can shape monitoring, drug preparation, postoperative placement, and rescue planning, but it has not been shown to improve outcomes.

*Evidence boundary: multidisciplinary assessment, disease phenotyping, selected invasive evaluation, and planned postoperative care are G; direct noncardiac perioperative risk estimates are D; the five-domain baseline and anticipated transition are H.*

### 6.2 Continuity of PH therapy and the pre-induction brief

Disease-targeted therapy should generally continue throughout the perioperative period. Continuous prostacyclin infusion must not be interrupted because abrupt withdrawal can cause rebound pulmonary vascular load and rapid RV failure. Oral or inhaled therapies require a documented plan when fasting, intubation, gastrointestinal dysfunction, or transfer prevents the usual route. Anticoagulation in chronic thromboembolic disease must be coordinated with surgical bleeding risk and neuraxial constraints.

The pre-induction brief should state the WHO group and hemodynamic type, baseline RV reserve, likely mechanism of deterioration, monitoring and treatment-continuity plans, location of selective inhaled vasodilator support, criteria for transesophageal echocardiography or pulmonary artery catheterization, the extracorporeal contact pathway, and the postoperative bed. The H-labeled phenotype vocabulary is optional; the operational plan is not.

*Evidence boundary: continuity of PH therapy and high-risk team planning are G; using the proposed phenotype name in the brief is H.*

### 6.3 Induction, airway management, and maintenance

Induction brings loss of sympathetic tone, vasodilation, myocardial depression, apnea, altered venous return, laryngoscopy, and positive-pressure ventilation. In a pressure-loaded RV, even brief systemic hypotension can reduce coronary driving pressure and start a low-output spiral. Small titrated anesthetic doses, adequate analgesia, avoidance of prolonged apnea and marked hypercapnia, and pressor readiness before vascular tone falls are more useful than reliance on a favored drug. No induction agent protects every patient; dose, rate, co-administered drugs, volume state, and immediate support determine the net effect.

Regional anesthesia can reduce airway and ventilation stress but may cause abrupt sympathectomy. General anesthesia permits controlled oxygenation and ventilation but introduces positive intrathoracic pressure and drug-related vasodilation. Choice should follow the procedure, anticoagulation, patient cooperation, respiratory reserve, and rescue capability. The framework does not claim superiority of one technique.

*Evidence boundary: avoidance of hypotension, hypoxemia, hypercapnia, and abrupt hemodynamic change is G/E; comparative evidence favoring one anesthetic technique in noncardiac PH surgery is insufficient.*

### 6.4 Ventilation as a hemodynamic intervention

Ventilation changes pulmonary vascular load and venous return. A reasonable starting approach uses a lung-protective tidal volume based on predicted body weight, the lowest inspired oxygen fraction that meets clinical need, individualized PEEP, avoidance of excessive plateau and driving pressures, and correction of severe hypercapnia or acidemia. These are starting conditions, not PH-specific outcome thresholds (32–34). Their value depends on the observed RV and flow response.

Recruitment or a PEEP adjustment is likely helpful when oxygenation and compliance improve without a fall in quantitative or directional flow, systemic pressure, or RV geometry. If the same adjustment raises airway pressure, enlarges the RV, lowers VTI or cardiac output, and reduces systemic pressure, overdistension or impaired venous return becomes more plausible. ETCO2 can support this interpretation only when ventilation, metabolism, and dead space are stable. Unilateral ventilation, pneumoperitoneum, positioning, bronchospasm, and auto-PEEP require the same contextual assessment.

*Evidence boundary: avoidance of reversible PVR triggers and lung-protective principles are G/E; a particular PEEP-flow response threshold or phenotype transition is H.*

### 6.5 Fluid, blood, and congestion

Hypotension should not automatically lead to large-volume resuscitation. When underfilling or hemorrhage is plausible, passive leg raising or a small rapidly reassessed fluid challenge can test that explanation. A reproducible rise in quantitative cardiac output or same-modality VTI without worsening RV size or venous congestion supports cautious replacement. Lack of a flow response, especially with rising CVP, RV dilation, septal shift, or adverse venous Doppler, argues against further fluid.

Blood products should address bleeding and oxygen-carrying needs, not serve as generic RV preload therapy. In congestive low flow, organ function may improve after unnecessary fluid is stopped, the arterial-to-venous organ gradient is preserved, excessive afterload is treated, contractility is supported when indicated, and decongestion is controlled. A patient can remain fluid responsive despite high RAP and RV dilation; responsiveness predicts a flow increment, not absence of congestion or net clinical benefit.

*Evidence boundary: response-guided fluid and avoidance of venous congestion are G/E; the 15% framework threshold and classification of a mixed congestive/preload-recruitable state are H.*

### 6.6 Vasoactive and pulmonary vascular support

Vasoactive therapy should match the observed physiology, and its value should be judged by the net response rather than a single pressure. Norepinephrine can increase pulmonary vascular tone, yet may provide net benefit after induction by restoring systemic vascular resistance and aortic pressure. Low-dose vasopressin is used as an adjunct in some settings, whereas higher doses may compromise coronary and tissue perfusion (35, 36). Experimental pressure-load studies show different pressure-flow trade-offs between norepinephrine and dobutamine (37). These data do not establish a universal first-line sequence for noncardiac PH surgery.

Dobutamine and milrinone can improve RV contractility and reduce PVR. Milrinone also reduces systemic vascular resistance and often requires vasopressor support; it is an inodilator, not a pressor. Dobutamine and epinephrine may produce tachycardia and increase myocardial oxygen demand. Epinephrine remains rational for refractory low output or cardiac arrest. Reassessment should include quantitative or directional flow, heart rate and rhythm, mean and diastolic pressure, RV geometry, oxygenation, lactate, and organ perfusion.

Inhaled nitric oxide and inhaled prostacyclin preferentially reach ventilated lung and can reduce RV afterload with less systemic vasodilation than intravenous therapy. Cardiac-surgical trials and meta-analyses show short-term hemodynamic improvement and broadly similar efficacy between agents (38–40). They do not establish routine prophylactic benefit for patient-important outcomes in noncardiac PH surgery. Rescue use should be linked to a plausible precapillary component and monitored for pulmonary edema in group 2 PH, worsening gas exchange in group 3 PH, systemic spillover, and rebound after abrupt withdrawal.

*Evidence boundary: general physiologic goals are G; comparative vasopressor, inotrope, and inhaled-vasodilator evidence is largely E; the phenotype-treatment pairings are H and should not be interpreted as a validated algorithm.*

### 6.7 Acute RV failure: parallel rescue before diagnostic iteration

Severe hypotension, a sudden fall in quantitative flow or ETCO2, hypoxemia, an abrupt CVP rise, new RV dilation or septal shift, worsening tricuspid regurgitation, or a new dysrhythmia should activate a predefined pathway. Tension pneumothorax, acute pulmonary embolism, hemorrhage, tamponade, severe bronchospasm, dynamic hyperinflation, coronary ischemia, and equipment or airway problems must be considered in parallel (41–49).

In an immediately life-threatening crisis, the instruction to apply one small intervention does not apply. Parallel actions take precedence: declare the emergency and mobilize PH and extracorporeal expertise; provide oxygen and verify the airway and circuit; correct extreme hypercapnia, acidemia, and excessive airway pressure; support systemic pressure and rhythm; stop plausible precipitating drugs or surgical triggers; obtain focused echocardiography and blood gas or electrolytes without delaying resuscitation; control hemorrhage or obstruction; and prepare selective pulmonary vasodilation or mechanical support when appropriate. Interventions should still be timestamped so the subsequent response can be interpreted.

Once immediate threats are controlled, the team can return to a smaller matched intervention and five-domain reassessment. Persistent low output despite treatment, rapidly escalating vasoactive requirements, or cardiac arrest warrants early mechanical-support activation at a capable center. Venoarterial extracorporeal membrane oxygenation can restore systemic perfusion and unload the RV as a bridge to recovery, diagnosis, pulmonary vascular intervention, or transplantation (9, 41–50). Timing, reversibility, center capability, and a defined bridge strategy determine its role.

*Evidence boundary: simultaneous correction of immediately reversible threats and early escalation are G/E; the two-track workflow and timestamped bundle-response record are H.*

### 6.8 Extubation, transfer, and recovery

Extubation and departure from the operating room are hemodynamic transitions. Residual anesthetic, pain, hypoventilation, fluid redistribution, dysrhythmia, anemia, and interruption of PH therapy can restart uncoupling. Readiness includes stable systemic perfusion and RV function, acceptable work of breathing and acid-base status, effective analgesia, manageable secretions, and a credible reintubation plan. Wakefulness and oxygen saturation alone are insufficient (51). Noninvasive respiratory support may reduce work of breathing, but excessive pressure can impair venous return or overdistend the lung.

Postoperative placement and monitoring duration should follow procedural stress and physiologic trajectory rather than a fixed timetable. Handover should state WHO group and hemodynamic type, baseline RV function, current physiology, medication schedule and prohibited interruptions, recent intervention-response pairs, crisis triggers, initial rescue actions, escalation contacts, and treatment limits.

*Evidence boundary: high-risk postoperative surveillance, treatment continuity, and structured transfer are G; the five-domain handover vector is H.*

## 7 A two-track closed-loop process

Figure 4 now separates two clinical tracks. In controlled instability, clinicians identify a provisional dominant mechanism, apply one small matched probe while minimizing nonessential competing changes, and reassess at a prespecified interval. In an immediately life-threatening crisis, a parallel rescue bundle addresses oxygenation and ventilation, systemic pressure and rhythm, major reversible causes, and escalation simultaneously. Both tracks converge on rapid reassessment; neither delays standard resuscitation.

**Figure 4:**
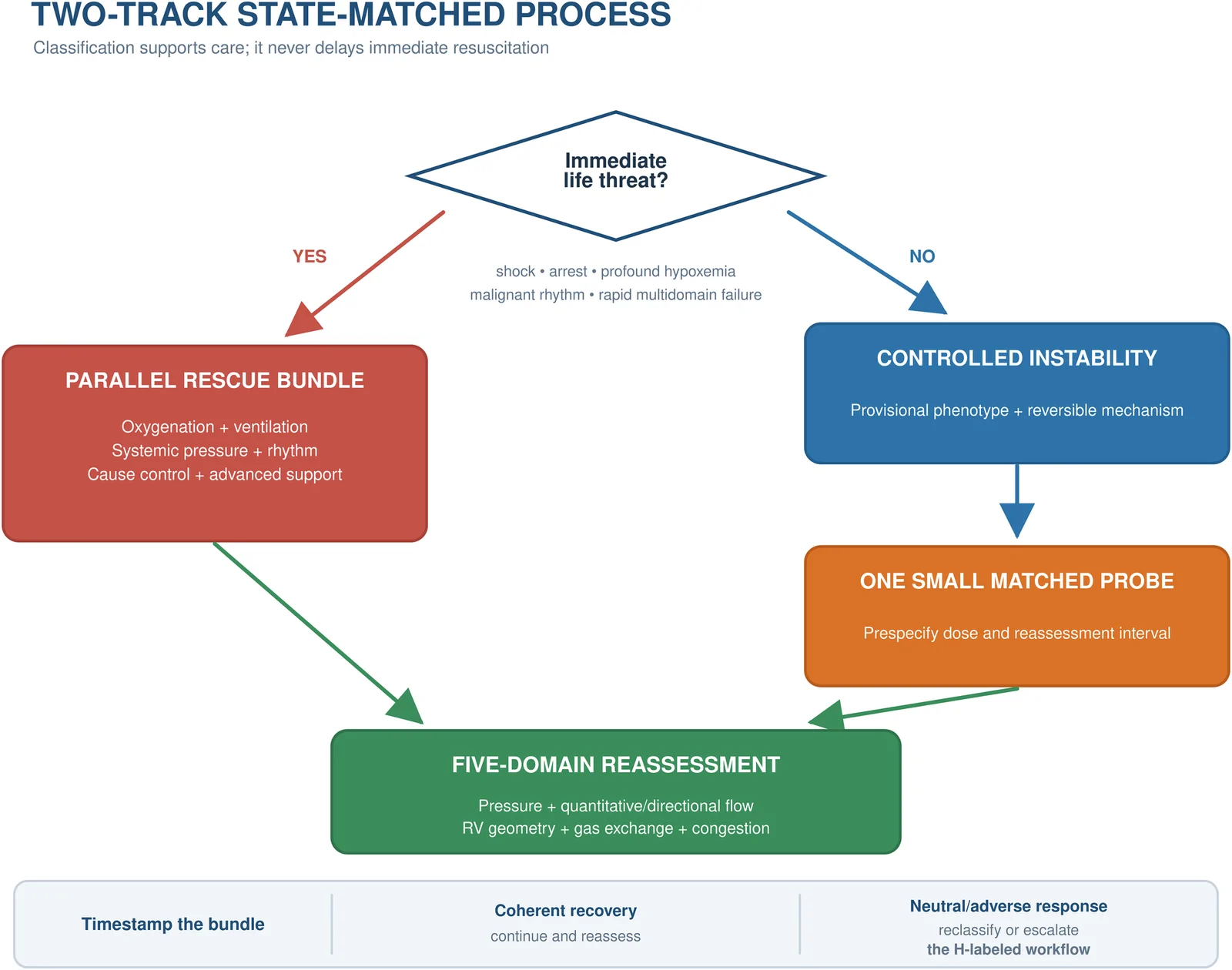
Two-track state-matched process. Controlled instability permits a provisional classification and one small matched probe followed by five-domain reassessment. Immediately life-threatening deterioration activates parallel rescue of oxygenation and ventilation, systemic pressure and rhythm, reversible causes, and advanced support. Both tracks converge on reassessment and reclassification or escalation when recovery is not coherent.

A timestamped intervention-response pair is the smallest auditable unit in the proposed framework. The record includes the trigger, certainty of classification, intervention or rescue bundle and dose, reassessment interval, and changes in systemic pressure, quantitative or directional flow, RV geometry, gas exchange, and congestion. Every variable is not required. The record should explicitly state when flow is supported only by downstream surrogates.

The process can be used at different monitoring levels. Arterial pressure, pulse oximetry, capnography, clinical perfusion, and focused echocardiography may be adequate in a lower-risk case. Active decompensation may justify invasive arterial pressure, central venous access, transesophageal echocardiography, pulmonary artery catheterization, or advanced cardiac-output monitoring. A device is useful when its result can change treatment; PH alone does not mandate every monitor.

## 8 Falsifiable predictions and validation program

### 8.1 Stage 1: classification manual and reliability

A multidisciplinary Delphi panel should refine the provisional rules without using outcome data. The resulting manual should define acceptable measurement windows, missing-data codes, mixed and indeterminate states, and how to judge independent corroboration. Clinicians blinded to outcomes should classify standardized vignettes and recorded episodes. Agreement should be reported for each phenotype and for the decision to remain indeterminate, using kappa and a prevalence-robust statistic such as Gwet's AC1 with 95% confidence intervals. Progression to clinical evaluation should require at least moderate reproducibility, prespecified as an overall coefficient of 0.60 or greater, with disagreement analysis used to simplify the model.

### 8.2 Stage 2: prospective multicenter observational cohort

Baseline variables should include WHO group and pre- versus postcapillary hemodynamics, WHO functional class, symptoms, natriuretic peptide, RV echocardiographic function, oxygen requirement, targeted therapy, prior decompensation, comorbidity, procedure type and urgency, expected physiologic stress, and center capability. Repeated snapshots should be recorded before induction; after induction and ventilation; after major changes in position, insufflation, bleeding, or vasoactive therapy; before extubation; at transfer; and during any deterioration. Each monitoring modality, signal quality, and intervention-response pair should be timestamped. Supplementary Table S3 provides the minimum dataset.

The primary clinical endpoint is adjudicated perioperative RV decompensation from induction through postoperative day 7. It requires new or clearly worsening objective RV dysfunction or pressure overload on echocardiography or invasive monitoring plus at least one clinical consequence: sustained low output or hypoperfusion requiring new or substantially escalated vasoactive or inotropic support; rescue inhaled pulmonary vasodilator specifically for RV failure; unplanned invasive ventilation or renal replacement attributable to RV failure; unplanned mechanical circulatory support; or cardiac arrest or death attributed to RV failure. Two adjudicators blinded to framework classification should review source data, with a third resolving disagreement. Secondary endpoints include severe hypotension, organ support, ICU and hospital length of stay, functional recovery, and survival at 30 and 90 days.

### 8.3 Comparator, analysis, and missing data

PAP alone is retained as a mechanistic secondary comparator, not the primary benchmark. The clinically meaningful base model should include PH group and hemodynamic type, WHO functional class, biomarkers, baseline RV function, oxygen requirement, prior decompensation, procedure type and urgency, comorbidity, and conventional perioperative pressure and rhythm variables. Incremental value of the time-updated five-domain representation should be tested beyond that base model using discrimination, calibration, Brier score, and decision-curve net benefit, with internal-external validation by center where feasible.

Repeated domain measurements require mixed-effects, multistate, or joint longitudinal-outcome models rather than treating observations as independent. Time-varying treatments should be modeled explicitly. Confounding and treatment-by-indication bias should be addressed with prespecified causal diagrams, time-dependent covariate adjustment, and sensitivity analyses such as inverse-probability weighting when assumptions are defensible. Center-level clustering should be handled with random effects or robust variance. Death before nonfatal outcomes should be analyzed according to the estimand with cause-specific and competing-risk approaches. Missingness should be described by signal and timepoint; multiple imputation may be used for plausibly missing covariates, but an unmeasured modality must not be imputed as a normal physiologic value. Complete-case and worst-case sensitivity analyses should be reported.

### 8.4 Prespecified predictions and failure criteria

Five predictions are retained but strengthened. First, the time-updated model should add meaningful information beyond the contemporary clinical base model, not merely beyond PAP. Second, falling PAP with falling quantitative or directional flow should carry greater risk than a similar PAP change with rising flow. Third, the composite context of low systemic pressure, high or rising RAP, impaired flow, and RV strain may identify a coherent response to pressure restoration; no isolated pressure-RAP threshold is proposed. Fourth, a preload challenge that raises venous pressure without increasing flow should predict subsequent congestion or organ dysfunction. Fifth, a phenotype-informed workflow should first improve process measures and time to coherent recovery before patient-centered benefit is claimed.

The model should be narrowed or rejected if classification is unreliable, if its trajectories do not add prognostic or decision information beyond the base model, if phenotype assignments do not modify intervention response, or if implementation adds delay or cognitive burden without improving process. Table 5 lists these tests and failure criteria.

**Table 5:** Staged validation questions, analyses, and failure criteria.

Domain	Question	Primary design/analysis	Finding that would challenge the framework
Reliability	Can clinicians classify states, including mixed/indeterminate?	Blinded vignette/episode classification; kappa and Gwet AC1; disagreement analysis	Overall agreement <0.60 or recurrent unsafe disagreement
Incremental prediction	Does the trajectory add value beyond current practice?	Base model: WHO group/type, functional class, biomarker, RV function, oxygen, history, procedure/urgency, comorbidity, conventional monitoring; add time-updated domains	No useful gain in calibration, Brier score, decision curve, or validated discrimination
Pressure-flow discordance	Does PAP down/flow down differ from PAP down/flow up?	Repeated quantitative/directional pairs linked to adjudicated outcomes	Outcome risk unrelated to directional concordance
Perfusion-pressure context	Does low pressure + high RAP + low flow + RV strain identify coherent pressor response?	Prespecified composite context; change in flow and RV geometry; no isolated delta threshold	No reproducible response or unacceptable adverse effects
Fluid/congestion interaction	Does venous-pressure rise without flow rise predict harm?	Standardized PLR/mini-fluid test; serial flow, geometry, venous and organ outcomes	No association with congestion or outcomes
Feasibility/process	Does the workflow improve care without delay or overload?	Treatment continuity, unassessed bolus rate, escalation delay, time to recovery, transfer completeness, burden	No process gain, excessive missingness, treatment delay, or cognitive burden

AC1, agreement coefficient 1; PAP, pulmonary artery pressure; PH, pulmonary hypertension; RAP, right-atrial pressure; RV, right ventricle.

### 8.5 Stage 3: pragmatic evaluation only after feasibility

A cluster-randomized or stepped-wedge evaluation should begin only after acceptable reliability, data completeness, and observational signal. The intervention would be a brief baseline vector, a state-certainty prompt, a two-track crisis override, a standardized intervention-response record, and structured transfer. It would not mandate a particular drug, PEEP, CVP, or diastolic pressure-RAP target. Initial endpoints should address protocol fidelity, time to coherent recovery, unassessed large fluid boluses, therapy interruptions, delayed escalation, and documentation burden. Clinical endpoints should follow only when effect size, contamination, and implementation burden are understood.

## 9 Worked clinical example

Table 6 follows a hypothetical patient with group 1 PAH through induction. The example is not clinical evidence and does not prescribe numeric targets. It shows that the five domains are not five separate algorithms: they are a structured way to update one working explanation. At baseline, the patient has high load with preserved flow. After induction, systemic and diastolic pressure and VTI fall while PAP changes little; the dominant working state becomes a perfusion-pressure/RV ischemic spiral. Because the patient is hypotensive but not yet in cardiac arrest or profound hypoxemia, a titrated vasopressor is used as a controlled probe while oxygenation and airway pressure are maintained. A rise in systemic pressure accompanied by recovery of VTI and less RV dilation supports the hypothesis; failure would trigger reclassification and escalation. If the same episode included profound hypoxemia, arrhythmia, or rapidly worsening shock, the parallel rescue track would replace sequential testing.

**Table 6:** Worked hypothetical example: transition across induction.

Time	Five-domain snapshot	Working interpretation	Action and reassessment
Pre-induction	PAP 65/30 mmHg; VTI 18 cm; MAP 82; stable RV size; no new congestion; SpO2 94%	Coupled high load	Prepare pressor and rescue; continue PH therapy; record baseline
2 min after induction	PAP 58/28; VTI 13 cm (−28%); MAP 48, diastolic 32; RAP rises 9 to 13; RV enlarges; oxygenation unchanged	Perfusion-pressure/RV ischemic spiral (H); PAP fall is not improvement	Controlled instability if no immediate life threat: titrated norepinephrine while maintaining airway/oxygenation; reassess promptly
After titrated pressor	MAP 68, diastolic 46; VTI 16 cm (+23% from nadir); RV size partly recovers; PAP 62/30	Working mechanism supported, not proven	Continue titration; examine rhythm, load, left filling, and congestion
If profound hypoxemia/arrhythmia develops	Rapidly worsening shock plus multiple simultaneous threats	Crisis override; classification secondary	Parallel oxygenation/ventilation, pressure/rhythm, trigger control, echo, and rescue activation; timestamp bundle and reassess

This hypothetical example is explanatory only and is not clinical evidence. MAP, mean arterial pressure; PAP, pulmonary artery pressure; RAP, right-atrial pressure; RV, right ventricle; VTI, velocity-time integral.

## 10 Discussion

Pulmonary artery pressure is incomplete and can be directionally misleading during perioperative RV-pulmonary arterial decompensation. That physiological fact is established. Pairing pressure with a quantitative or directional flow signal and then checking RV geometry, systemic perfusion, congestion, and gas exchange can make familiar paradoxes easier to recognize: PAP may fall during circulatory collapse; a vasopressor may improve flow even if PAP rises; fluid may reduce organ perfusion by raising venous pressure; PEEP may help through recruitment or harm through overdistension and impaired venous return; and pulmonary vasodilation may relieve precapillary load while destabilizing postcapillary filling or gas exchange.

The framework's contribution is organizational and empirical, not discovery of new physiology. Existing AHA, ISHLT, BJA, ESC/ERS, ASE, and POQI guidance already addresses disease phenotype, RV assessment, treatment continuity, reversible PVR triggers, systemic-pressure support, judicious fluid, and rescue planning (2, 7–9, 16, 17, 29–31). The H-labeled additions are a longitudinal pressure-flow representation, provisional operational phenotype rules, explicit mixed and indeterminate states, an auditable intervention-response unit, and predictions with failure criteria. These additions should be useful only if they improve decisions beyond current multidomain clinical practice.

The revised model is intentionally simpler at the bedside than its full conceptual description. Figure 1 is the rapid pressure-flow screen. Figure 2 explains the five-domain data structure. Table 4 supplies the optional working label. Figure 4 governs what happens next and includes a crisis override. The worked example shows how these pieces describe one evolving patient rather than competing layers. RV geometry can legitimately contribute to both reserve and current congestion, but it should be counted once within a classification episode.

Important limitations remain. Direct comparisons of anesthetic technique, ventilator settings, fluid targets, vasoactive sequences, and prophylactic inhaled vasodilators across PH groups are sparse. Most noncardiac perioperative data are observational; many treatment inferences come from physiology, acute RV failure, cardiac surgery, or critical care. The evidence-source labels do not constitute a formal GRADE assessment. Flow measures differ in validity and time resolution, and the proposed 15% change rule may be too small or too large for particular methods. ETCO2, venous oxygen saturation, lactate, urine output, and clinical signs cannot substitute for measured flow when ventilation, metabolism, hemoglobin, oxygen consumption, or organ reserve changes.

Phenotypes overlap, and classification may be unreliable during rapidly evolving shock. The systemic diastolic pressure-RAP difference is not coronary perfusion pressure, has no validated threshold, and should not direct therapy in isolation. WHO group and pre- versus postcapillary physiology can reverse the benefit-harm balance of pulmonary vasodilation or fluid. A negative pragmatic trial could reflect a flawed model, poor implementation, or both, so fidelity and burden must be measured. The narrative search lacked a contemporaneous screening log and formal duplicate selection, which limits reproducibility despite the supplied full search strings and retained-source categories.

The full perioperative continuum offers a useful test environment because the same baseline vulnerability is exposed to fasting and medication interruption, induction, airway management, ventilation, bleeding, pain, emergence, transfer, and recovery. A useful model should improve continuity as well as crisis care, without delaying parallel resuscitation when the patient is unstable. Functional recovery and return to baseline activity belong beside short-term hemodynamic endpoints.

## 11 Conclusion

Perioperative PH is a dynamic interaction between the right heart, pulmonary circulation, systemic pressure, venous return, and oxygen delivery. The fall of PAP during low-output RV failure is established physiology. The proposed five-domain framework does not replace contemporary guidance; it organizes familiar information into a longitudinal pressure-flow trajectory, provisional and explicitly unvalidated phenotype rules, and timestamped intervention-response records. Quantitative flow estimates should be distinguished from stroke-volume trends and downstream surrogates, and WHO group must modify every interpretation. Controlled instability may permit a small matched diagnostic-therapeutic probe, whereas immediately life-threatening deterioration requires parallel resuscitation. Clinical use as an algorithm should await reproducible classification and incremental value beyond current risk assessment, RV function, biomarkers, procedure characteristics, and conventional monitoring.
