# Supplementary material for: Pressure-flow discordance in perioperative pulmonary hypertension: a mechanism-based review and provisional five-domain right-heart model for noncardiac surgery
Source: Front Med (Lausanne). 2026 Sep 28;13:1972194. doi: 10.3389/fmed.2026.1972194 (PMC13656009; doi:10.3389/fmed.2026.1972194)
Supplement: Supplementary file 1 [file Table1.docx]

Supplementary Material

This supplement describes the evidence-search documentation, claim provenance, proposed minimum dataset, and provisional evaluation rules for the five-domain perioperative right-heart framework. It contains no patient-level dataset, completed validation study, or observed treatment-effect estimate. All new classification and evaluation rules are hypotheses for prospective testing.

# Supplementary Table S1 Evidence search documentation

The manuscript reports literature coverage through 31 July 2026. This is the stated coverage cutoff, not a verified date on which each search was executed. The supplied files contain one principal PubMed query and a list of targeted concepts; they do not contain database exports, dated repository logs, or a contemporaneous de-duplication and screening record.

The query below is transcribed from the supplied supplement. The structured search blocks on the next page are a proposed reproducible rerun strategy developed for this revision. They must not be represented as the exact historical searches or as newly executed searches.

# S1A Principal query retained from the source material

(("Hypertension, Pulmonary"[Mesh] OR "pulmonary hypertension"[tiab] OR "pulmonary arterial hypertension"[tiab]) AND ("Perioperative Care"[Mesh] OR perioperat*[tiab] OR anesthesia[tiab] OR anaesthesia[tiab] OR "noncardiac surgery"[tiab] OR "non-cardiac surgery"[tiab]) AND ("Ventricular Dysfunction, Right"[Mesh] OR "right ventricular"[tiab] OR "right heart failure"[tiab] OR hemodynamic*[tiab] OR haemodynamic*[tiab] OR ventilation[tiab] OR fluid*[tiab] OR congestion[tiab] OR vasopressor*[tiab] OR inotrope*[tiab] OR vasodilator*[tiab] OR "extracorporeal membrane oxygenation"[tiab]))

| **Source or method** | **Reported scope** | **Documentation boundary** |
| --- | --- | --- |
| PubMed/MEDLINE | Database inception through 31 July 2026 | Historical run date, retrieved count and exported records not supplied. |
| Society and guideline repositories | ESC/ERS, AHA/ACC, ISHLT, ASE, POQI and relevant journal sources | Repository-specific queries and access dates not supplied. |
| Backward citation screening | Pivotal statements and reviews | No dated citation-screening log supplied. |
| Selection and synthesis | Iterative narrative selection; no pooled effect estimate | No duplicate systematic screening or retrospectively reconstructed PRISMA counts. |

Eligibility follows main-text Section 2: adult PH definitions, noncardiac perioperative evidence, RV physiology and measurement, and explicitly identified indirect evidence. Pediatric, obstetric and isolated congenital-heart studies are excluded except for indispensable general physiology. Evidence-source labels identify provenance rather than GRADE certainty.

# Supplementary Table S1 Proposed reproducible rerun strategy

S1B. Use the following PubMed search-history blocks. Run #1–#7 separately; then execute each combination shown below with the date restriction #7. The added date restriction is a publication-date filter, not proof of the historical availability or indexing date of a record. These blocks have not been executed as part of this supplement preparation.

| **Block** | **PubMed syntax** |
| --- | --- |
| #1 PH | "Hypertension, Pulmonary"[Mesh] OR "pulmonary hypertension"[tiab] OR "pulmonary arterial hypertension"[tiab] |
| #2 Perioperative care | "Perioperative Care"[Mesh] OR perioperat*[tiab] OR anesthesia[tiab] OR anaesthesia[tiab] OR "noncardiac surgery"[tiab] OR "non-cardiac surgery"[tiab] |
| #3 RV physiology | "Ventricular Dysfunction, Right"[Mesh] OR "right ventricular failure"[tiab] OR "right heart failure"[tiab] OR "ventricular arterial coupling"[tiab] OR "ventricular-arterial coupling"[tiab] |
| #4 Pressure and flow | ("pulmonary artery pressure"[tiab] OR "pulmonary arterial pressure"[tiab] OR "pulmonary vascular resistance"[tiab]) AND ("cardiac output"[tiab] OR "stroke volume"[tiab] OR "velocity time integral"[tiab] OR "velocity-time integral"[tiab]) |
| #5 Interventions | ventilation[tiab] OR PEEP[tiab] OR fluid*[tiab] OR congestion[tiab] OR vasopressor*[tiab] OR inotrope*[tiab] OR vasodilator*[tiab] OR "extracorporeal membrane oxygenation"[tiab] |
| #6 Disease modifiers | "left heart disease"[tiab] OR postcapillary[tiab] OR "post-capillary"[tiab] OR "lung disease"[tiab] OR thromboembolic[tiab] OR hypoxia[tiab] |
| #7 Date limit | ("1800/01/01"[Date - Publication] : "2026/07/31"[Date - Publication]) |

Noncardiac perioperative PH: #1 AND #2 AND #7. RV physiology and coupling: #3 AND (#1 OR #2 OR "critical care"[tiab]) AND #7. Pressure–flow assessment: #4 AND (#1 OR #3) AND #7. Intervention and rescue: #5 AND (#1 OR #3) AND #7. Disease-specific modifiers: #1 AND #6 AND #7.

For any future rerun, retain the exact query, platform, execution date, retrieved count, export filename and eligibility decisions. Record society/repository name, document title, version, URL or DOI, and access date separately. Do not manufacture unavailable historical counts. Source numbers in S2 refer to the accompanying main manuscript bibliography.

# Supplementary Table S2 Evidence provenance and limits

G = guideline, consensus or scientific statement; D = direct noncardiac perioperative PH clinical data; E = extrapolated physiology, measurement, critical-care or cardiac-surgery evidence; H = authors’ hypothesis. Multiple labels distinguish established components from proposed extensions. They do not rank certainty or imply formal GRADE assessment.

| **Claim** | **Label** | **Main refs** | **Interpretive boundary** |
| --- | --- | --- | --- |
| Perioperative PH risk | D; G | 10–15; 7–9 | Risk varies with PH group, reserve, surgery and urgency; associations do not validate the new model. |
| Disease group and treatment context | G | 1–9 | Precapillary, postcapillary, lung-disease and thromboembolic mechanisms require different interpretation. |
| PAP depends on load and flow | E; G | 20–21; 1–2 | Falling PAP can accompany failing output. The longitudinal representation itself is H. |
| RV reserve and coupling | E; G | 22–30; 31–32 | Noninvasive surrogates remain load-, rhythm- and image-dependent; reserve is not one cutoff. |
| Ventilation and pulmonary load | E; G | 33–35; 7–9, 32 | Recruitment and overdistension have different effects; no universal PH-specific PEEP target follows. |
| Fluid responsiveness and congestion | E; G | 23–25, 42–47; 7–9, 32 | A flow response does not establish net benefit or exclude congestion. |
| Systemic pressure and vasoactive support | E; G | 36–38, 42–47; 7–9 | Diastolic pressure minus RAP is not measured RV coronary perfusion pressure. The composite phenotype is H. |
| Inhaled pulmonary vasodilators | E; G | 39–41; 7–9 | Cardiac-surgical evidence does not establish routine noncardiac prophylaxis; group-specific hazards remain. |
| Advanced rescue and VA-ECMO | E; G | 42–51; 9 | Center expertise, reversibility and bridge strategy limit transferability. |
| Five domains and five provisional phenotypes | H | Main Sections 4–5; Tables 1, 3 | No completed validation. Mixed and indeterminate states are legitimate outcomes. |
| 15% flow change and corroboration rules | H | Main Section 3.3; Table 3 | Feasibility rules only; use a larger threshold when local measurement error requires it. |
| Two-track workflow and prospective evaluation | H | Main Sections 7–8; Table 6 | Must show reliability, incremental value and feasibility without delaying rescue. |

The indirect treatment literature and each proposed phenotype–treatment pairing must remain distinguishable. A physiologically coherent response supports a working explanation; it neither proves causation nor establishes patient-centered benefit.

# Supplementary Table S3 Proposed minimum dataset

This is a data dictionary for a future observational study, not a report of enrolled patients. Record raw values, units, timestamps, acquisition method and signal quality before deriving changes or assigning a phenotype. No invasive monitor should be placed solely to complete the proposed record.

| **Record domain** | **Required elements when available** | **Units or recording rule** |
| --- | --- | --- |
| Identity and timing | Pseudonymous patient, center and episode IDs; date/time; perioperative phase; intervention and measurement timestamps | ISO 8601 time with time zone; repeated records linked to the same patient. |
| Disease and baseline reserve | WHO group; pre/postcapillary type; diagnostic source/date; functional class; BNP or NT-proBNP; RV size/function; oxygen and PH therapy; prior decompensation | Retain assay units and source dates. Unknown type remains unknown. |
| Procedure and exposure | Urgency, operation, anesthetic technique, position, pneumoperitoneum, one-lung ventilation, blood loss, coexisting disease, rescue capability | Record event onset and changes rather than only case-level summaries. |
| Pressure and rhythm | Systemic systolic/diastolic pressure and MAP; PAP components; RAP/CVP; PAWP when available; heart rate/rhythm | mmHg; beats/min; zero/reference level and ventilation context. Distinguish invasive from estimated pressure. |
| Forward flow | CO; stroke volume; LVOT VTI; LVOT diameter/area and HR if echo CO is derived | L/min, mL/beat, cm; acquisition method, calibration, beats averaged and quality. VTI alone is not CO. |
| RV and venous findings | RV/LV size, septal shape, TAPSE, FAC, strain, TR, venous Doppler and congestion signs | Retain units, image window, method and availability; avoid inferring normality from absent imaging. |
| Ventilation and oxygen delivery | Tidal volume and predicted body weight; PEEP; plateau/driving pressure; auto-PEEP; FiO2, SpO2, PaO2, PaCO2, pH, ETCO2; Hb; SvO2/ScvO2; lactate | Keep original units; specify sampling site and timing. Capture confounders of ETCO2 and venous saturation. |
| Intervention and response | Trigger; proposed phenotype/certainty; drug/fluid/blood/ventilator change; dose, route, start/stop; concurrent treatments; reassessment interval | Allow a rescue bundle. Record pre/post values, percentage change, adverse effects and subsequent reclassification. |
| Outcomes and completeness | RV endpoint criteria, adjudication and onset; organ support; hypotension; ICU/hospital stay; death and recovery at 30/90 days | Code not measured, unreliable, not applicable and unavailable separately; missing is never physiologic normality. |

CO, cardiac output; CVP, central venous pressure; FAC, fractional area change; Hb, hemoglobin; LVOT, left-ventricular outflow tract; MAP, mean arterial pressure; PAP, pulmonary artery pressure; PAWP, pulmonary artery wedge pressure; RAP, right-atrial pressure; RV, right ventricle; TAPSE, tricuspid annular plane systolic excursion; TR, tricuspid regurgitation; VTI, velocity-time integral.

# Supplementary Note S1 Provisional classification manual

These rules implement the unvalidated definitions in main-text Table 3. Use paired measurements from a prespecified, clinically meaningful window. Calculate percentage change as 100 × (follow-up − baseline) / baseline when the baseline is valid and nonzero. A ≥15% CO or same-modality VTI change is a provisional measurement rule; use a larger threshold if required by local repeatability.

Classify flow evidence as quantitative when technically valid serial CO estimates are available, directional when a reliable same-modality stroke-volume trend is available, and supportive or indeterminate when only downstream surrogates are available. The last category comprises the supportive, oxygen-balance and downstream-organ rows of main-text Table 2. Changes in method, ventilation, rhythm or image quality may invalidate comparison.

## Coupled high load

High or rising load/PAP with stable or rising flow, plus at least two of stable systemic perfusion, stable RV geometry and preserved oxygen delivery. New RV dilation or falling flow requires reassessment.

## Effective preload limitation

A ≥15% flow rise during a clinically indicated preload maneuver under stable measurement conditions, plus at least two of plausible volume or venous-return loss, small/normal RV or no geometric worsening, and no progressive congestion. A positive response with congestion is a mixed state.

## Afterload-driven RV–PA uncoupling

A ≥15% flow fall and new/worsening RV dilation or septal flattening, with an acute load trigger and at least one independent perfusion or congestion change. PAP may rise early or fall late.

## Perfusion-pressure or RV ischemic spiral

Falling systemic/diastolic pressure with a ≥15% flow fall, plus at least two of high/rising RAP, RV dilation, an ischemic ECG/echo clue or relevant dysrhythmia. The pressure–RAP difference has no validated standalone threshold.

## Congestive low flow

A ≥15% flow fall with high/rising venous pressure or congestion and no ≥15% flow response to an appropriate preload maneuver, plus at least one of RV dilation/TR, abnormal venous Doppler or organ dysfunction. Do not perform a potentially harmful challenge simply to establish this label; record untested responsiveness and uncertainty.

## Mixed and indeterminate states

Record all supported mechanisms when criteria overlap; designate a dominant state only when evidence supports that judgment. Record indeterminate when core measurements are missing, unreliable or confounded. Document the reason and next feasible observation.

Corroboration should represent distinct information rather than repeated expressions of one signal: MAP and diastolic pressure alone are not two independent domains. Record supporting and conflicting observations. A working label must change when the response contradicts the mechanism. Classification must never delay emergency treatment.

# Supplementary Note S2 Observation schedule and response record

| **Time point** | **Observation window** |
| --- | --- |
| T0 | Preoperative stabilized baseline; retain the date of earlier tests. |
| T1 | Immediately before induction. |
| T2 | Within 5 minutes after induction and ventilation, when feasible. |
| T3 | After major positioning, insufflation, one-lung ventilation or a material ventilator change. |
| T4 | Before and after a clinically indicated fluid, blood, vasoactive, inotropic or inhaled-vasodilator intervention. |
| T5 | At decompensation and after rescue steps; chart actual times retrospectively if needed to avoid delaying care. |
| T6–T7 | Before emergence or planned postoperative ventilation; at transfer of care. |
| T8 | 6–24 hours postoperatively; continue event surveillance through postoperative day 7. |
| T9 | Hospital discharge. |
| T10–T11 | 30-day and 90-day survival and functional recovery. |

# Intervention and response recording

Record the trigger; immediate-life-threat assessment; provisional phenotype and certainty; pre-intervention measurements and their quality; intervention or rescue bundle with doses, route and timing; concurrent changes; planned reassessment interval; actual follow-up time; available five-domain responses; adverse effects; and the subsequent decision. Reassessment timing must match the intervention and urgency; one universal interval is not proposed.

During controlled instability, a small matched intervention may help examine the working mechanism while other nonessential changes are minimized. During immediately life-threatening deterioration, simultaneous rescue takes precedence. Record the bundle rather than attributing its response to a single component.

# Coherent improvement

The provisional definition requires improvement in the targeted domain, stable or favorable change in at least two independent additional domains, and no prespecified safety deterioration. A change in PAP alone is insufficient. If required domains are unavailable, record the response as incompletely assessable rather than assume coherent recovery. When flow is represented only by surrogates, state this explicitly.

# Reliability and analysis safeguards

A pilot manual should be finalized before outcome evaluation. Outcome-blinded assessors should record all five labels, mixed states and indeterminate states. Report agreement and uncertainty, including kappa and Gwet’s AC1 with 95% confidence intervals. The manuscript proposes ≥0.60 overall agreement as a feasibility gate, not an established standard.

Use patient-linked repeated-measure analyses, account for center and time-varying treatment, and test added value beyond the contemporary clinical base model in Section 8.3. Describe missingness by signal and time point; do not convert an unmeasured modality into normal physiology. Prediction does not establish causal treatment benefit.

# Supplementary Note S3 Clinical endpoint and safety outcomes

# Primary endpoint and adjudication

The proposed primary clinical endpoint is adjudicated perioperative RV decompensation from induction through postoperative day 7, consistent with main-text Section 8.2. It requires new or clearly worsening objective RV dysfunction or pressure overload on echocardiography or invasive monitoring, together with at least one of the following clinical consequences:

• Sustained low output or hypoperfusion requiring new or substantially escalated vasoactive or inotropic support.

• Rescue inhaled pulmonary vasodilator specifically for RV failure.

• Unplanned invasive ventilation or renal replacement attributable to RV failure.

• Unplanned mechanical circulatory support.

• Cardiac arrest or death attributed to RV failure.

Two adjudicators blinded to framework classification should review source data, with a third resolving disagreement. Record the objective RV finding, consequence, timing, attribution and alternative explanations. A treatment escalation without qualifying objective RV evidence does not automatically satisfy this endpoint. Missing evidence should be recorded as insufficient for adjudication, not as absence of an event.

Before enrollment, a prospective protocol must specify duration and dose-change criteria for “sustained” and “substantially escalated,” acceptable imaging/hemodynamic criteria, adjudication rules and follow-up after early discharge. These numerical definitions are not established by this review. The earlier 30-day composite is not retained as the primary endpoint.

# Secondary and safety outcomes

Secondary outcomes include severe hypotension; unplanned organ support; ICU and hospital length of stay; survival at 30 and 90 days; and functional recovery relative to preoperative status. Report components separately rather than relying only on the primary composite.

Core safety outcomes are pulmonary edema; worsening oxygenation or ventilation–perfusion matching; new tachyarrhythmia; myocardial ischemia; severe systemic hypotension; fluid accumulation with organ venous congestion; acute kidney or hepatic injury; unplanned ventilation or ICU admission; mechanical circulatory support; rebound after interruption or withdrawal of inhaled or continuous PH therapy; and death. Use prospectively chosen accepted definitions where available, recording onset, severity and attribution. Do not attribute all perioperative organ injury to RV failure.

# Data and interpretation boundaries

No new patient or animal data are supplied. The worked clinical example in main-text Table 5 is hypothetical. The proposed dataset, classification rules, endpoints and workflow are materials for future validation, not results of a completed study. Any future participant research requires its own ethics and governance arrangements. The present review does not establish the clinical efficacy or safety of the framework.
